# Verbal and Physical Abuse Against Nurses Working in Hospitals and Health Centers in Buraidah, Saudi Arabia

**DOI:** 10.7759/cureus.31792

**Published:** 2022-11-22

**Authors:** Fatimah Sayed, Awatif Mansoor Alrasheeday, Bushra Alshammari, Afaf Alonazi, Ahmed Alharbi, Nahla Abdullah Almotairi, Unaib Rabbani

**Affiliations:** 1 College of Nursing, University of Hail, Hail, SAU; 2 Family Medicine Academy, Qassim Health Cluster, Buraidah, SAU

**Keywords:** saudi arabia, physical, verbal, abuse, violence, nurses

## Abstract

Background

Hospital staff, especially nurses, face violence of various forms in the workplace. This study aimed to assess the burden of verbal/physical abuse against nurses and their attitude towards such events in Buraidah, Saudi Arabia.

Methods

A cross-sectional study was conducted among nurses working in three public sectors and two private sector hospitals, and five primary healthcare facilities in Buraidah. Data was collected using a structured online questionnaire, disseminated among nurses through nursing departments of participating facilities. Data were analyzed using SPSS version 21.0.

Results

A total of 369 nurses participated in the survey, with a mean age of 34 (±6.9) years. About two-thirds, 68% (250) of respondents, had experienced at least one verbal/physical abuse incident during one year. The most common perpetrators were patients (44.4%). Sixteen percent of participants reported taking no action against the incident. Those working in private facilities were 80% less likely to experience abuse than those in public facilities, adjusted OR 0.20 (95% CI: 0.08-0.47).

Conclusions

There was a high burden of verbal/physical abuse against nurses, and they, therefore, suffer from disturbing memories. However, a little more than half do not officially report it to the managers, with only a small fraction seeing some action taken. Occupational health practitioners should take action to improve the policy and procedures related to workplace violence (WPV) in healthcare facilities. Further research is needed to characterize incidents to understand the patterns and develop interventions for the prevention of such events.

## Introduction

Violence in the workplace is a growing phenomenon. The burden of the problem is largely unknown because of the gross under-reporting of workplace violence (WPV) [1]. All the working sectors are affected by WPV; however, there are certain sectors in which workers are at higher risks of violence, such as the health sector. Workers in professions (e.g., health) that deal with people in distress often assume WPV as a regular part of their job [2]. WPV takes many forms, and it could be physical or non-physical. This is expressed in many ways, such as physical assault, homicide, verbal abuse, bullying, sexual harassment, and threat [3]. Verbal abuse is a form of violence that is most common. This does not leave a visible scar, but it does hurt the victim to the core and damages their self-being [4]. Verbal abuse may be in the form of words, tone, or manner that ridicules, threatens, blames, or disrespects others [5].

Violence in the workplace can affect the morale and performance of the workers. Studies have shown that WPV in the health sector is associated with low job satisfaction, intention to switch the job, increased job turnover, fear, anger, sadness, disturbing memories related to a violent incident, symptoms of post-traumatic distress and depression, and poor functioning which may further cause medical errors [6-11].

Workplace abuse (verbal or physical) against nurses is an essential issue as this affects the nurses in terms of their performance, patient safety, organization, and overall healthcare delivery system. Globally several studies have been carried out to measure the burden and assess the patterns of violence against nurses [9, 12-15]. However, in Saudi Arabia, literature on this subject is scarce, and most of the studies are concentrated in Riyadh and focused on healthcare workers in general [16-20]. To the best of our knowledge, no study has been published from the Qassim region to characterize verbal abuse against nurses. Therefore, this study aimed to assess the burden and predictors of verbal and physical abuse against nurses and to explore nurses' responses to the violence against them in Buraidah, Saudi Arabia.

Conceptual framework

For this study, we used Roy's model of adaptation. This model was originally developed by Sister Callista Roy in 1976. This has been widely used in understanding adaptation behavior to various circumstances in nursing practice. For example, several studies have used this model to understand the violence against nurses and their adaptation to cope with this problem [21, 22]. This model helps understand the stimuli that trigger the violent/abusive behavior against the nurse and the victim's responses to the incident and its impact on them. The nurse in our study is the adaptive system that responds positively or negatively to the stimuli that are an incident of verbal abuse in a given environment. Stimuli could be a physician, senior nurse, peer, patient, or visitor. This environment could be an ED, consultation room, ward, operation theater, vaccination room, or assessment room. Exposure to the incidence of verbal abuse causes neural, chemical, and endocrine responses within the adaptive system (Nurse), which results in an automatic, unconscious response such as physiological responses. These emotions help seek relief from the stimuli (verbal abuse). Another adaptive sub-system is the cognator system which responds by processing information, learning, judgment, and solving the problem. Coping mechanisms or strategies are responses that could be innate or acquired and help in solving the issue or improving feelings. Examples include sharing with friends or families and smoking etc. The impact or long-term effects of verbal abuse are displayed through adaptive behaviors of modes. These include physiological (physical response), role function (expectations about the roles or acts of individuals in one occupation towards others), self-concept (personal feelings and beliefs about one's self), and interdependence (working and personal relationships). Finally, positive behaviors could be adaptive and help nurses improve their self-esteem, maintain integrity, and develop personal and professional skills. On the other hand, there could be ineffective behaviors by being unable to end the verbal abuse, which leads to decreased integrity and self-esteem and increased adverse outcomes.

## Materials and methods

Study design and setting

A cross-sectional study was carried out from October to November 2021 in five primary healthcare centers and five hospitals (three public and two private sectors) in Buraidah, Qassim. Buraidah is the capital of the Qassim region, with half of the region's population living here.

Study population

This study's population included all the nurses working in the primary health care centers and hospitals of Buraidah, Qassim region. Nurses were eligible to participate if registered with Saudi Commission for Health Specialties and had been working in the facility for at least one year. We excluded undergraduate and trainee nurses as they work under supervision and may not independently be involved in patient care.

Sample size calculation

The sample size was calculated using WHO software for sample determination in health studies. Previous studies in Saudi Arabia showed that WPV was between 24.7% and 57%. Therefore, we used 57% for calculating the sample as this would give the largest sample being closest to 50%. At a 95% confidence level, a bound-on error of 5%, and an expected prevalence of 57%, the required sample size was 377 participants.

Sampling technique and procedure

We selected five primary health care centers out of 43 total in Buraidah by simple random sampling using Microsoft Excel. There are four public sector hospitals in Buraidah, out of which three were included, and one which is for mental health was excluded as the inclusion of participants from that hospital might overestimate the burden of violence. There are two hospitals in the private sector, and both were included as study sites. Convenience sampling was used to recruit participants from hospitals and primary healthcare centers. There are currently 5,980 nurses working in public sector healthcare facilities in Qassim. Out of these, 4776 (80%) are in hospitals while 1204 (20%) are working in primary health care centers. Participants were recruited online. The nursing directors of selected facilities were approached, and a questionnaire link was shared with them to disseminate among all the nursing staff of their facilities.

Operational definition of workplace violence

The workplace violence definition used in this study is also taken from the study done by Al-Shamlan NA et al. [18], which is "Intended use of physical or psychological force resulting in fear of harm or other negative consequences to the targeted individuals or groups, including behaviors that humiliate, degrade, or otherwise indicate a lack of respect for the dignity and worth of an individual."

Study instrument and data collection procedure

Data was collected using an online structured questionnaire. After taking written permission, the questionnaire was adopted and modified [18]. This questionnaire was derived from the WPV in the health sector questionnaire, developed as a joint program of the International Labor Office, WHO, International Council of Nursing, and Public Service International [23]. The questionnaire had two main sections. The first section was about the socio-demographic and professional characteristics of the participating nurses. This includes variables related to age, gender, marital status, qualification, and work experience. The second part was about WPV. Participants were asked if they had experienced verbal or physical abuse in the last twelve months before the survey. Those who responded 'yes' were asked further questions about the perpetrator (patient, attendant or staff, and gender), place of incident (inside or outside the facility), response to the incident (reporting, sharing, seeking help, or no action etc.), effects of the incident on them (disturbing memories, avoiding thinking, being more alert), handling of the incident (reporting, action taken, consequences for perpetrator), and satisfaction with the handling process. The first page of the questionnaire contained information about the research, informed consent, and the researcher's contact information. 

Data analysis

Data were analyzed using SPSS version 21.0. Descriptive analysis was done to calculate the frequency and proportions of categorical variables and means with SDs for continuous variables. The prevalence of verbal and physical abuse was determined through frequency and proportions. The Chi-squared test was used to look for the differences in verbal abuse prevalence with respect to gender, nationality, qualification, and work experience. In addition, logistic regression analysis was carried out to assess the factors associated with verbal and physical abuse against nurses. Crude and adjusted ORs were calculated using univariate and multivariate logistic regression analysis, respectively. A p-value less than 0.05 was considered significant for all inferential statistics.

## Results

A total of 369 nurses participated in the survey. The mean age of the participants was 34 (±6.9) years. The majority (90.5%) were female. About 39% (143) were non-Saudis. The majority, 83% (306), was working in hospital settings. Ninety-two percent (339) were in a public sector facility. The mean years of work experience were 9.35 (±6.6) years. Near three-quarters, 73% (270), worked either the evening or night shift (Table 1).

**Table 1:** Socio-demographic and professional characteristics of the nurses (n=369). PHC: Primary healthcare.

Variable	% (n)	
Age		
Mean (SD)	34.18 (6.90)	
Gender		
Female	90.5 (334)	
Male	9.5 (35)	
Marital Status		
Never married	26.6 (98)	
Ever married	73.4 (271)	
Nationality		
Saudi	38.8 (143)	
Non-Saudi	61.2 (226)	
Type of facility		
Hospital		82.9 (306)
Primary care center		14.6 (54)
Other		2.4 (9)
Nature of facility		
Public		91.9 (339)
Private		8.1 (30)
Place of work		
Emergency		14.9 (55)
ICU		17.1 (63)
OPD-Hospital		15.7 (58)
OPD-PHC		11.9 (44)
Administration		9.5 (35)
In-patient		24.9 (92)
Operation room		6.0 (22)
Years of experience		
Means (SD)		9.35 (6.6)
Work in the evening or night shift		
Yes		73.2 (270)
No		26.8 (99)
Number of staff present in the same shift/place		
One to three		42.8 (158)
Four to six		19.2 (71)
Seven to nine		10.8 (40)
More than nine		27.1 (100)

The proportion of staff highly worried about abuse was 12% (44). Regarding the presence of reporting procedures for a violent incident, 66.7% (246) thought there were procedures. About 51% (188) said there is an encouragement to report WPV. About 74% (272) said "yes" regarding the intention to report an incident of abuse. The most common entity to report was manager/employer, 83.5% (294), followed by colleagues 9.7% (34) (Table 2).

**Table 2:** Attitudes of nurses towards physical/verbal violence (n=369).

Variable	% (n)
		Worriedness about violence at workplace	
Not worried at all	32.0 (118)
Little worried	22.8 (84)
Somewhat worried	23.0 (85)
Worried	10.3 (38)
Highly Worried	11.9 (44)
Are there procedures for reporting violence?	
Yes	66.7 (246)
No	33.3 (123)
Encouragement to report workplace violence	
Yes	50.9 (188)
No	26.6 (98)
May be	22.5 (83)
If you are abused, will you report it?	
Yes	73.7 (272)
No	4.6 (17)
May be	21.7 (80)
Who will you report (n=352)	
Manager/Employer	83.5 (294)
Colleagues	9.7 (34)
Association	1.1 (4)
Family/Friends	2.8 (10)
Other	2.8 (10)

About two-thirds, 68% (250) of respondents, had experienced at least one incident of verbal/physical abuse during the last 12 months. The average number of incidents was 2.69 (±2.63) in the last year. The most common perpetrators were patients, 44.4% (111), and males, 58% (114). The majority of the verbal/physical abuse incidents took place inside the facility, 91% (228).

The most frequent actions taken after the incident were reporting to the senior staff, 47.6% (119). Sixteen percent (40) of participants reported that they took no action against the incident. Around 18% (46%) pretended as if it had never happened. Other actions included asking the perpetrator to stop 39.2%, sharing with a colleague 34.4%, sharing with friends/family 33.6%, seeking counseling 25%, seeking help from an association 18.8%, and moving from job 12%.

Table 3 shows after effects and handling of the incident of verbal/physical on nurses. About a quarter (26%) of the victims had disturbing memories after the incident. About 11% (27) and 13% (33) avoided thinking about the incident often and always, respectively. About 23% (58) were often or always watchful after the incident.

**Table 3:** Effects of verbal/physical abuse on nurses and their perception about incident handling (n=250).

Variable	% (n)
Disturbing memories	
Never	22.4 (56)
Rarely	26.4 (66)
Sometimes	24.8 (62)
Often	16.4 (41)
Always	10.0 (25)
I avoid thinking about incident	
Never	19.2 (48)
Rarely	30.8 (77)
Sometimes	26.0 (65)
Often	13.2 (33)
Always	10.8 (27)
I have been more watchful	
Never	21.6 (54)
Rarely	26.4 (66)
Sometimes	28.8 (72)
Often	10.0 (25)
Always	13.2 (33)
I think everything was useless	
Never	13.6 (34)
Rarely	28.0 (70)
Sometimes	34.8 (87)
Often	13.2 (33)
Always	10.4 (26)
Do you think incident could have been prevented?	
No	11.6 (29)
Yes	88.4 (221)
How could it have been prevented	
Understanding the offender	59.6 (149)
Through people and security	35.6 (89)
Avoiding shift work	44.0 (110)
Was any action taken	
Yes	18.4 (46)
No	79.2 (198)
May be	2.4 (6)
Who took the action (n=46)	
Manager	60.9 (28)
Employer	2.2 (1)
Police	37.0 (17)
Consequences for offender	
None	21.7 (10)
Verbal warning	21.7 (10)
Handed over to police	4.3 (2)
Prosecuted	17.4 (8)
Don’t know	34.8 (16)
Supported by the management	
Counselling	23.2 (58)
Opportunity to speak about incident	22.8 (57)
Other	36.4 (91)
Satisfaction with the incident handling	
Very dissatisfied	24.5 (61)
Dissatisfied	46.6 (116)
Neutral	14.5 (36)
Satisfied	11.2 (28)
Very satisfied	3.2 (8)

A great majority, 88% (221), who experienced verbal/physical abuse thought that incident could have been prevented. The most common perceived preventive strategy was understanding the offender, 59.6% (149). Regarding the action taken for the incident, only 18.4% said "Yes." The action takers were managers 61% (28) and police 37% (17). About 22% (10) said there was no consequence for the offender and the same proportion reported a verbal warning only. The support provided to the victim included counseling 23.2% (58) and the opportunity to speak about the incident 22.8% (57). Nearly three-fourths (177) of the victims were dissatisfied with the incident handling.

We found no significant difference in the age and gender of those who experienced the incident and those who did not experience it. Similarly, there were no differences in the prevalence of abuse incidents with respect to marital status and nationality. The prevalence of abuse was significantly higher in public sector facilities, 70.8%, as compared to private facilities, 33.3% (p-value <0.001). There were no significant differences regarding the type of facility, place of work, years of experience, shift work, and the number of staff on the same shift (Table 4).

**Table 4:** Distribution of incident of verbal/physical abuse according to socio-demographic and professional characteristics of nurses (n=250). ¥ Mann-Whitney U test p-value. PHC: Primary healthcare.

Characteristic	Verbal/physical abuse		P-value
	No % (n)	Yes % (n)	
Age			
Mean (SD)	34.0 (6.56)	34.3 (7.06)	0.683
Gender			
Female	31.1 (104)	68.9 (230)	0.158
Male	42.9 (15)	57.1 (20)	
Marital Status			
Never married	32.7 (32)	67.3 (66)	0.921
Ever married	32.1 (87)	67.9 (184)	
Nationality			
Saudi	30.1 (43)	69.9 (100)	0.476
Non-Saudi	33.6 (76)	66.4 (150)	
Type of facility			
Hospital	32.0 (98)	68.0 (208)	0.299
Primary care center	29.6 (16)	70.4 (38)	
Other	55.6 (5)	44.4 (4)	
Nature of facility			
Public	29.2 (99)	70.8 (240)	<0.001
Private	66.7 (20)	33.3 (10)	
Place of work			
Emergency	20.0 (11)	80.0 (44)	0.246
ICU	34.9 (22)	65.1 (41)	
OPD-Hospital	25.9 (15)	74.1 (43)	
OPD-PHC	34.1 (15)	65.9 (29)	
Administration	34.3 (12)	65.7 (23)	
In-patient	37.0 (34)	63.0 (58)	
Operation room	45.5 (10)	54.5 (12)	
Years of experience			
Means (SD)	8.99 (6.4)	9.52 (6.68)	0.373^¥^
Work in the evening or night shift			
Yes	31.5 (85)	68.5 (185)	0.602
No	34.3 (34)	65.7 (65)	
Number of staff present in the same shift/place			
01-Mar	37.3 (59)	62.7 (99)	0.126
04-Jun	29.6 (21)	70.4 (50)	
07-Sep	37.5 (15)	62.5 (25)	
More than 9	24.0 (24)	76.0 (76)	

In the multivariate regression analysis, the only significant predictor of verbal/physical abuse was the nature of the facility. Those working in private facilities were 80% less to experience abuse as compared to those in public facilities, adjusted OR 0.20 (95% CI: 0.08-0.47). Other factors, such as place of work and number of staff, which were significant in the univariate analysis, were not significant in the multivariate model after adjusting for possible confounding effects of other variables (Table 5).

**Table 5:** Logistic regression analysis of the factors associated with verbal/physical abuse among nurses. PHC: Primary healthcare.

Characteristic	Verbal/physical abuse		P-value
	No % (n)	Yes % (n)	
Age			
Mean (SD)	34.0 (6.56)	34.3 (7.06)	0.683
Gender			
Female	31.1 (104)	68.9 (230)	0.158
Male	42.9 (15)	57.1 (20)	
Marital Status			
Never married	32.7 (32)	67.3 (66)	0.921
Ever married	32.1 (87)	67.9 (184)	
Nationality			
Saudi	30.1 (43)	69.9 (100)	0.476
Non-Saudi	33.6 (76)	66.4 (150)	
Type of facility			
Hospital	32.0 (98)	68.0 (208)	0.299
Primary care center	29.6 (16)	70.4 (38)	
Other	55.6 (5)	44.4 (4)	
Nature of facility			
Public	29.2 (99)	70.8 (240)	<0.001
Private	66.7 (20)	33.3 (10)	
Place of work			
Emergency	20.0 (11)	80.0 (44)	0.246
ICU	34.9 (22)	65.1 (41)	
OPD-Hospital	25.9 (15)	74.1 (43)	
OPD-PHC	34.1 (15)	65.9 (29)	
Administration	34.3 (12)	65.7 (23)	
In-patient	37.0 (34)	63.0 (58)	
Operation room	45.5 (10)	54.5 (12)	
Years of experience			
Means (SD)	8.99 (6.4)	9.52 (6.68)	0.373^¥^
Work in the evening or night shift			
Yes	31.5 (85)	68.5 (185)	0.602
No	34.3 (34)	65.7 (65)	
Number of staff present in the same shift/place			
1-Mar	37.3 (59)	62.7 (99)	0.126
4-Jun	29.6 (21)	70.4 (50)	
7-Sep	37.5 (15)	62.5 (25)	
More than 9	24.0 (24)	76.0 (76)	

## Discussion

This study is one of its kind from the Qassim region to assess the attitude towards and burden of WPV, its characteristics, and risk factors among nurses working in hospitals and primary care set-ups. The study found that about 55% of the nurses were either not or a little worried about verbal/physical abuse. Only two-thirds, 66.7%, reported having procedures for handling such incidents. The one-year prevalence of verbal/physical abuse was found to be 68%. The most common perpetrator was patients (44.4%). Sixteen percent of the abuse victims took no action at all. A little more than a quarter of the victims had disturbing memories after the incident. Eighty-eight percent of the victims thought that event was preventable. In 18.4% of the cases, action was taken, and only 14.4% of these were satisfied with the way the incident was handled. The only significant factor associated with verbal/physical abuse was the nature of the facility.

Physical and verbal abuse are common problems in healthcare settings. Nurses are often affected by these violent incidents. Studies have shown that attitude and knowledge about WPV and its procedures affect the vulnerability of nurses to such incidents. This study found that 66.7% of nurses were aware of the reporting procedures for verbal/physical violence. This is higher than reported in Bahrain, where 60% were aware of policies [24]. This is an important finding which calls for hospital and healthcare facility administrations to widely disseminate the policies and procedures related to reporting and handling workplace violence. This should be made part of the job orientation programs of every healthcare organization. In this study, a little more than half were little or not worried at all about verbal or physical abuse, while 45% were moderately to highly worried about such incidents. This needs to be addressed as this might affect their mental health, job satisfaction, and performance. This can be addressed by clear policies and procedures and staff support in terms of their security and an optimal number of staff.

The burden of verbal/physical abuse is high in all settings. This study found that 68% of the nurses were victims of verbal or physical abuse in the last year. This is comparable to estimates from the USA, 70% verbal and 50% physical [13]. However, our estimates are lower than Bahrain's 78% [24], USA's 76% [25], Iran's 87% [26], and Turkey's 80% [27], while higher than Jordan's 55% [12] and Ghana's 52% verbal [9]. In the local context, this study reported a higher prevalence of abuse as compared to Riyadh 47% [16], 54.3% [28], Eastern Province 30.7% [18], Alhassa 28% [20], Abha 57.5% [17], while lower than another study from Riyadh 83% [19], which was conducted in EDs of three hospitals. This comparison of the reported burden of verbal/physical abuse across local and international studies should be interpreted cautiously as there are wide variations in the study settings (hospital, primary care, ED), study tools and definitions of outcomes, health system, and population characteristics. All these factors can lead to variations in the estimation of the problem. Nonetheless, this study shows that two-thirds of the nurses have been affected by such incidents, which is very high and needs the attention of the hospital and local health administration.

Studying the perpetrators' characteristics is vital to understand them and educating staff about such characteristics to be more careful while providing care. This study found that the most common perpetrators were patients 44.4%, relatives of patients 34.4% and staff members 11.2%. This finding is similar to a previous study conducted in Riyadh, where patients 52%, relatives 30%, and staff 7.8% were common perpetrators [18]. A similar pattern was reported in a study by Alyaemni A and Alhudaithi H in Riyadh. Another study from Riyadh, however, reported that patient relatives were the most common perpetrators, 80% [29]. This study, however, was conducted among emergency medical service staff who are likely to deal with severe patients who may be unconscious; therefore, relatives are the ones to communicate with care providers. In this study, the male gender as the perpetrator was 58% lower than Al-Hassa, KSA, where 95% of perpetrators were males [20].

Abuse in any form against nurses has been underreported. This is due to various reasons, ranging from non-existent or ineffective policies and procedures to a lack of trust in such procedures [12, 24, 28]. In this study, only 47% reported to managers or supervisors, while 16% took no action, and 18% pretended it had never happened. This is similar to a study from Riyadh, where 40.7% reported the incident [28]. Another study from the Eastern province, KSA, found that 86.7% did not report it officially [18]. However, nurses often share the incident with colleagues, friends, and family members. Reasons for not reporting the incident in various studies were found to be a lack of trust, reporting being useless, feeling of shame, and not considering the incident as necessary [12, 28, 29]. This practice needs to be changed by encouraging reporting of even minor incidents so that the actual burden and impact on nurses can be ascertained and preventive strategies can be implemented.

WPV has been associated with negative effects on the mental and physical health of the staff, poor performance, job dissatisfaction, and turnover [11]. The effect of such incidents could be short-term to long-term, depending on the nature of the abuse. In this study, a quarter of nurses had disturbing memories always, and a number tried to avoid thinking about the event and be more watchful. A study from Turkey reported that among those who had experienced verbal and physical abuse, 65% and 59%, respectively, had disturbed mental health [30]. A study from Al-Hassa, KSA, reported that even higher proportions of victims were bothered (96.7%), suspicious (86%), and watchful (46%) [20]. In this study, a significant majority (88.4%) of the victims assumed that the incident was preventable. This could have been prevented by understanding offenders (60%), people, and security (35.6%) and avoiding shift work (23%). A study from Riyadh reported that 69% of the victims thought that incident was preventable [18]. It has been reported that shortage of staff, lack of security, free movement of people in the facilities, and language barriers are common precipitating factors of WPV [28]. This calls for the administration to implement a policy of visitors, improve security and deploy an optimal number of staff in each unit and shift.

Handling such incidents is an essential factor that affects the prevention in the future and reporting by the victims. This study found that among those reported, the action was taken in only 18.4% of the cases. This shows management's seriousness toward protecting their staff and preventing WPV. However, the satisfaction with the matter was handled was low as only 14.4% were satisfied. A study by Al-Shamlan NA et al. in the Eastern province reported that only 1.8% of the victims were satisfied with the handling procedures of their hospitals. This indicates that the existing systems of violence incident handling are poor and do not perform well. This could lead to discouragement among staff to report such incidents and intention to move from the job. The risk factors of verbal and physical abuse against healthcare workers reported in the literature are inconsistent and vary from setting to setting [12, 14, 15, 25, 28]. In this study, the burden of verbal/physical abuse was higher in the public sector than in private facilities. This finding is consistent with results from a study conducted in Riyadh, where nurses working in public sector healthcare facilities reported a higher number of abuses than private workers [28]. There is wide variation in the distribution and associated factors of verbal/physical abuse in literature.

Strengths and limitations

This study is one of its kind to estimate the burden and characterize the verbal/physical abuse against nurses in the Qassim region. We used a validated questionnaire in Arabic and English to capture local and expatriate nurses. However, certain limitations must be considered while interpreting the results of this study. Firstly, the survey link was shared by nursing directors in their staff group; therefore response rate could not be ascertained. Secondly, recall and reporting biases could affect the responses of the participants. However, we assume this to have minimal impact on the validity of the results as the data collection form was anonymized, and the nursing supervisors or directors were not involved in data handling. Thirdly, the study was powered only for the prevalence of verbal/physical abuse, and it may not have sufficient power to detect differences across categories and associations. Therefore, the results of these inferential analyses should be interpreted cautiously.

## Conclusions

This study found a high burden of violence against nurses in healthcare settings. There was gross under-reporting of such violent acts against nurses. These events cause mental trauma and bad memories among the victims. There were deficiencies in the handling of incidents of violence against nurses. These findings have implications for occupational health practices in healthcare facilities as occupational health practitioners can play a vital role in improving the reporting of violence against nurses through simplifying procedures and avoiding blaming the victim approach. Healthcare facilities should ensure the safety of workers and strengthen the procedures to handle events of violence against healthcare workers. Furthermore, occupational health practitioners should also take part in investigations of such events to discover the factors that could help prevent violence against nurses in the future. There is a need to research on a large scale to characterize the problem further and develop effective interventions to prevent violence against healthcare workers.
